# CpGmotifs: a tool to discover DNA motifs associated to CpG methylation events

**DOI:** 10.1186/s12859-021-04191-8

**Published:** 2021-05-26

**Authors:** Giovanni Scala, Antonio Federico, Dario Greco

**Affiliations:** 1 Department of Biology, University of Naples “Federico II”, 80126 Naples, Italy; 2 Faculty of Medicine and Health Technology, Tampere University, 33014 Tampere, Finland; 3 Finnish Hub for Development and Validation of Integrated Approaches (FHAIVE), Tampere University, 33014 Tampere, Finland; 4 Institute of Biotechnology, University of Helsinki, 00014 Helsinki, Finland

**Keywords:** DNA methylation, DNA motifs, DNA methylation signature, Transcription factors, R-Shiny

## Abstract

**Background:**

The investigation of molecular alterations associated with the conservation and variation of DNA methylation in eukaryotes is gaining interest in the biomedical research community. Among the different determinants of methylation stability, the DNA composition of the CpG surrounding regions has been shown to have a crucial role in the maintenance and establishment of methylation statuses. This aspect has been previously characterized in a quantitative manner by inspecting the nucleotidic composition in the region. Research in this field still lacks a qualitative perspective, linked to the identification of certain sequences (or DNA motifs) related to particular DNA methylation phenomena.

**Results:**

Here we present a novel computational strategy based on short DNA motif discovery in order to characterize sequence patterns related to aberrant CpG methylation events. We provide our framework as a user-friendly, shiny-based application, CpGmotifs, to easily retrieve and characterize DNA patterns related to CpG methylation in the human genome. Our tool supports the functional interpretation of deregulated methylation events by predicting transcription factors binding sites (TFBS) encompassing the identified motifs.

**Conclusions:**

CpGmotifs is an open source software. Its source code is available on GitHub https://github.com/Greco-Lab/CpGmotifs and a ready-to-use docker image is provided on DockerHub at https://hub.docker.com/r/grecolab/cpgmotifs.

**Supplementary Information:**

The online version contains supplementary material available at 10.1186/s12859-021-04191-8.

## Background

DNA methylation aberration has been associated with several pathological conditions and, more recently, recognized as an important therapeutic target [1]. One of the most interesting aspects related to DNA methylation regards the ability of a CpG site to be more or less susceptible to changes in its methylation status. In fact, the propensity of CpG sites to de novo methylation is notably heterogeneous over the whole genome [2]. The reasons for such a discrepancy have been widely investigated. For instance, it has been demonstrated that a quantitative relationship exists between the DNA sequence composition and the stability of the associated methylation patterns [3, 4]. While this quantitative relationship has been extensively studied [5, 6], the existence of qualitative features between a particular DNA sequence and its methylation status has not been fully explored. The concept of DNA motif can be used to associate CpG methylation changes to their DNA context [1]. The usage of DNA motifs allows researchers to easily explore families of closely related DNA sequences that are over-represented in a set of sequences of interest. Such an approach has been used in [7] to identify DNA motifs that regulate DNA methylation, in [8] to identify transcription factor-based epigenetic signatures in neural cells and in [1] DNA sequences associated to changes in methylation patterns in different cancer types. The concept of methylation related motifs analysis has already been implemented in several other tools in the literature. For example, SEMplMe [9] uses motif analysis to predict the effects of methylation patterns measured in a BS experiment within binding site regions derived from a ChIP-seq experiment. Also, MotifMaker [10] identifies motifs associated with SMRT output methylation sequences. Here, we propose a strategy aimed to identify DNA motifs over-represented in a set of aberrantly methylated DNA sequences, obtained from a microarray-based DNA methylation experiment. This allows the user to pinpoint DNA motifs which are prone to undergo aberrant methylation in a certain biological condition. Moreover, our tool predicts transcription factor binding sites (TFBS) encompassing the identified motifs hence facilitating the exploration of possible consequences of specific DNA methylation patterns on the downstream gene transcriptional regulation. In this way, our tool supports the user in the biological interpretation of the results of a DNA methylation experiment. We developed CpGmotifs, an interactive tool based on a Graphical User Interface (GUI) implemented in R-Shiny. CpGmotifs provides a seamless and straightforward analytic flow to researchers investigating DNA motifs associated with methylation changes.

## Implementation

### Input

The input is constituted by one or more foreground sets of CpGs of interest (TargetCpGs) and one background set (BackCpGs). Each TargetCpG set is provided as a tab separated file containing 3 mandatory columns and an optional column. Each TargetCpG set consists of a table where each CpG is annotated with its methylation status (hyper-methylated or hypo-methylated). The background set represents the universe, typically defined as the whole set of tested CpGs, from which the TargetCpG set is derived. A flanking region size around each CpG site is used to retrieve the flanking sequences of each CpG site in the reference genome. The choice of the region size depends on various factors that are related to (1) the particular biological question, (2) the chosen motif search algorithm, and (3) the computational resources. In the next step, the tool retrieves the target and background flanking sequence sets using the BSgenome R package (https://www.bioconductor.org/packages/release/bioc/html/BSgenome.html).

### Motif discovery

The target and the background sequence sets are then provided as the input to a de novo DNA motif search tool. For this task, we make use of the DREME [11] tool from the MEME suite, which provides in output enriched DNA motifs. Once the enrichment analysis is completed, statistically significant motifs, along with corresponding information, are collected. In particular, for each significant motif, the following information is retrieved by parsing the DREME output xml file in R: (1) the motif consensus sequence; (2) the motif enrichment e-value; (3) the number of sequences carrying the motif in the TargetCpGs-seqs set; (4) the number of sequences not carrying the motif in the TargetCpGs-seqs set. For each discovered motif, the tool retrieves the corresponding list of CpGs containing the motif in their flanking sequence. This is accomplished by retrieving from the TargetCpGs sequences set all the motif consensus sequences reported in the DREME output. Once this set is obtained, the tool annotates the discovered motifs with the following additional fields (as reported in Additional file 1: Table S1):

- The number of hyper-methylated CpGs in the supporting set;
- The number of hypo-methylated CpGs in the supporting set;
- The methylation ratio of the supporting set (number of hyper-methylated CpGs/number of hypomethylated CpGs);
- The methylation unbalance p-value, computed by using an enrichment test over the contingency matrix built with hyper(hypo)-methylated CpGs in the supporting set and hyper(hypo)-methylated CpGs in the background set.

### Motif characterization

Once the annotated motifs are retrieved, it is possible to compare the obtained motifs among different experimental conditions or the different methylation trends, in order to get an insight on the specificity of the phenomenon under consideration. By using the *pairwiseAlignment* function from the Biostrings R package (https://bioconductor.org/packages/release/bioc/html/Biostrings.html), the tool computes the distance between two motifs using their consensus sequence (provided as IUPAC codes) and a nucleotide substitution matrix obtained through the *nucleotideSubstitutionMatrix* function from the Biostrings R package. The obtained similarity scores are normalized in the interval [0–1] using min-max normalization and the corresponding distance is computed as its complement to 1. Once the distance between all pairs of motifs has been obtained, a distance matrix is computed and used to perform clustering analysis. Hierarchical clustering is applied and the results are represented by means of a dendrogram adorned with methylation information from each motif by using the functions provided by the dendextend R package [12].

### Regulatory annotation

**Fig. 1:**
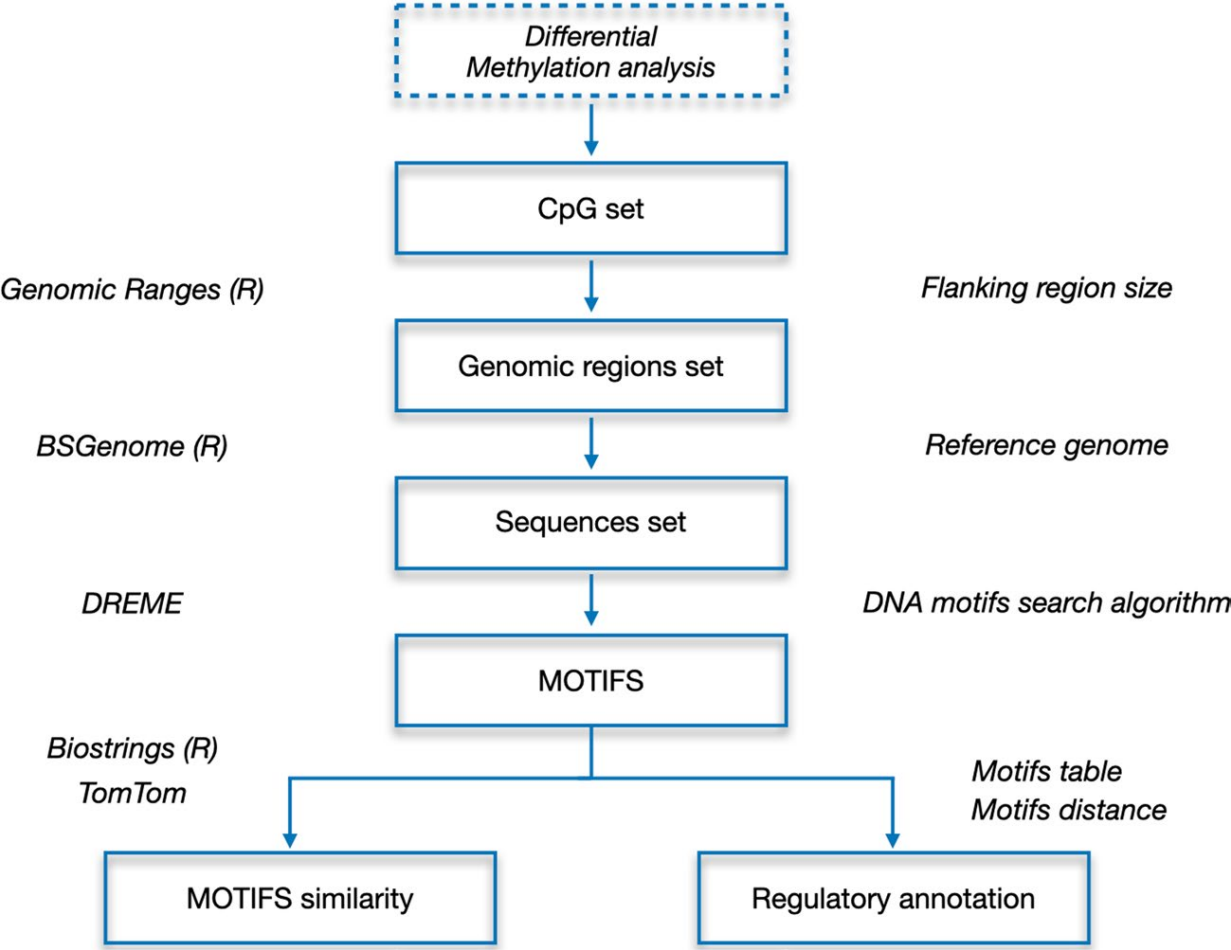
Search and analysis of DNA motifs related to CpG methylation events. Each box represents one of the intermediate steps of the proposed analysis. For each arrow, the required parameters are reported on the right side, while the employed tools to carry out the step are shown on the left side

DNA motifs are primarily used to search for affinities between binding sites of molecules interacting with the DNA, including transcription factors (TFs). In this setting, they can be used to study which TFs show affinity with the discovered motifs, and how their function is modulated by the methylation status of the associated CpG. CpGmotifs interrogates public databases of known motifs such as JASPAR [13], using the Tomtom [14] tool from the MEME suite and the obtained motifs as input. A list of highly similar motifs along with significance p-value and the corresponding binding proteins is returned and associated with each discovered motif (Fig. 1).

**Fig. 2:**
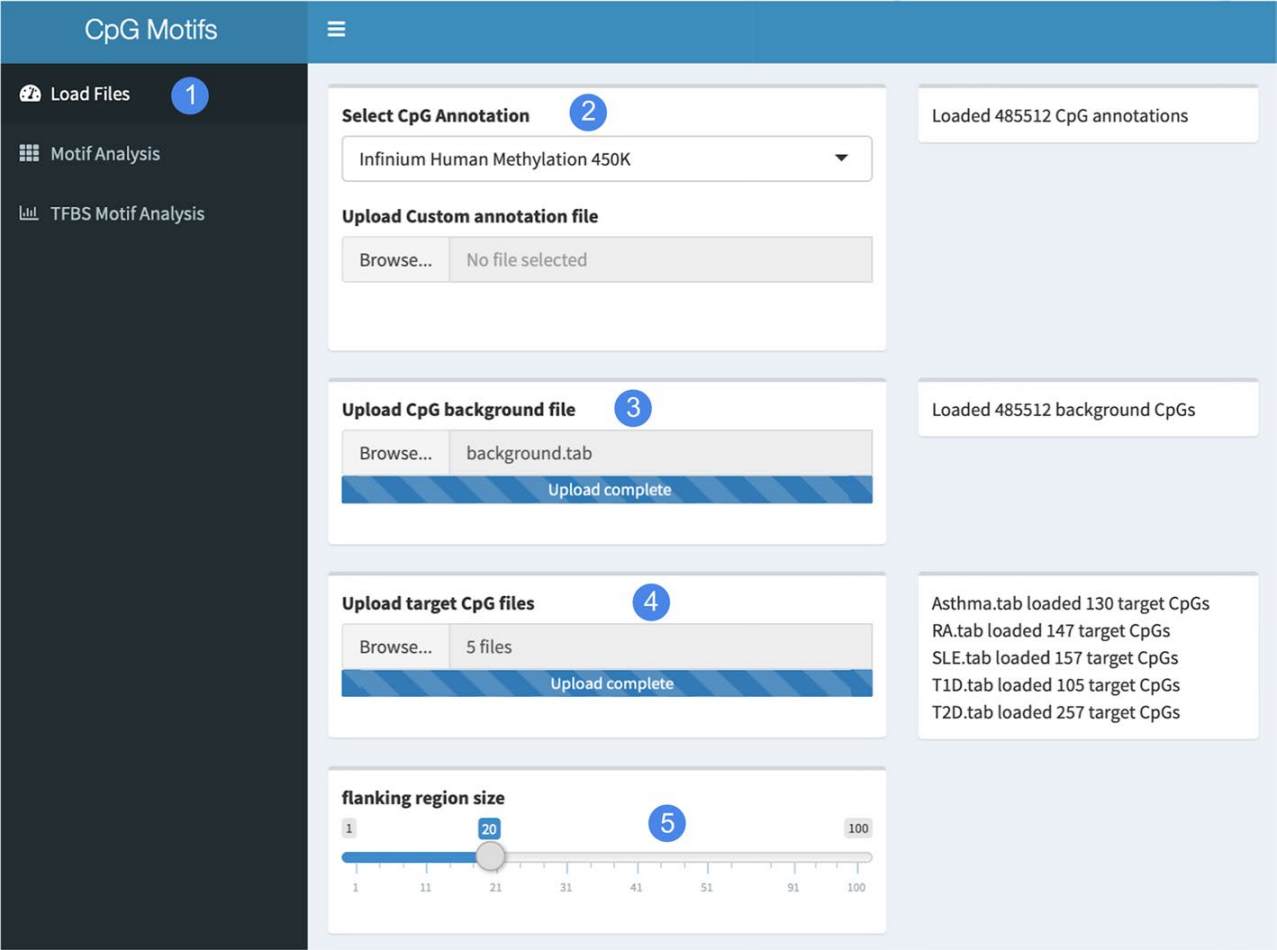
Files loading interface. Input files loading interface of the CpGmotifs tool

### CpGmotifs tool

The CpGmotifs tool is built as a user-friendly and intuitive interface in order to allow the users to have a smooth experience throughout the entire analysis. A typical CpGmotifs analysis goes through three main steps, performed by using the three tabs located on the left side of the main interface. The first step consists of loading the required files and annotations, as shown in Fig. 2 (1). In particular, the user first selects the proper annotation (2) that is needed to associate genomic coordinates (and associated sequences) to CpG IDs. The user can then choose between the Illumina Infinium Human Methylation 450K (https://support.illumina.com/array/array_kits/infinium_humanmethylation450_beadchip_kit.html) and the Infinium MethylationEPIC (https://www.illumina.com/products/by-type/microarray-kits/infinium-methylation-epic.html) annotations or, alternatively, load a custom hg19 annotation. Next, the user uploads a CpG background file (3) containing all the CpG sites representing the enrichment background, including their genomic location. Similarly, the user provides one or more target CpG files (4) containing the CpGs of interest for the motif enrichment analysis. All of the uploaded files must be in tab separated format. Finally, the user may set the size of the CpG flanking region where the tool will search for enriched motifs (5).

**Fig. 3:**
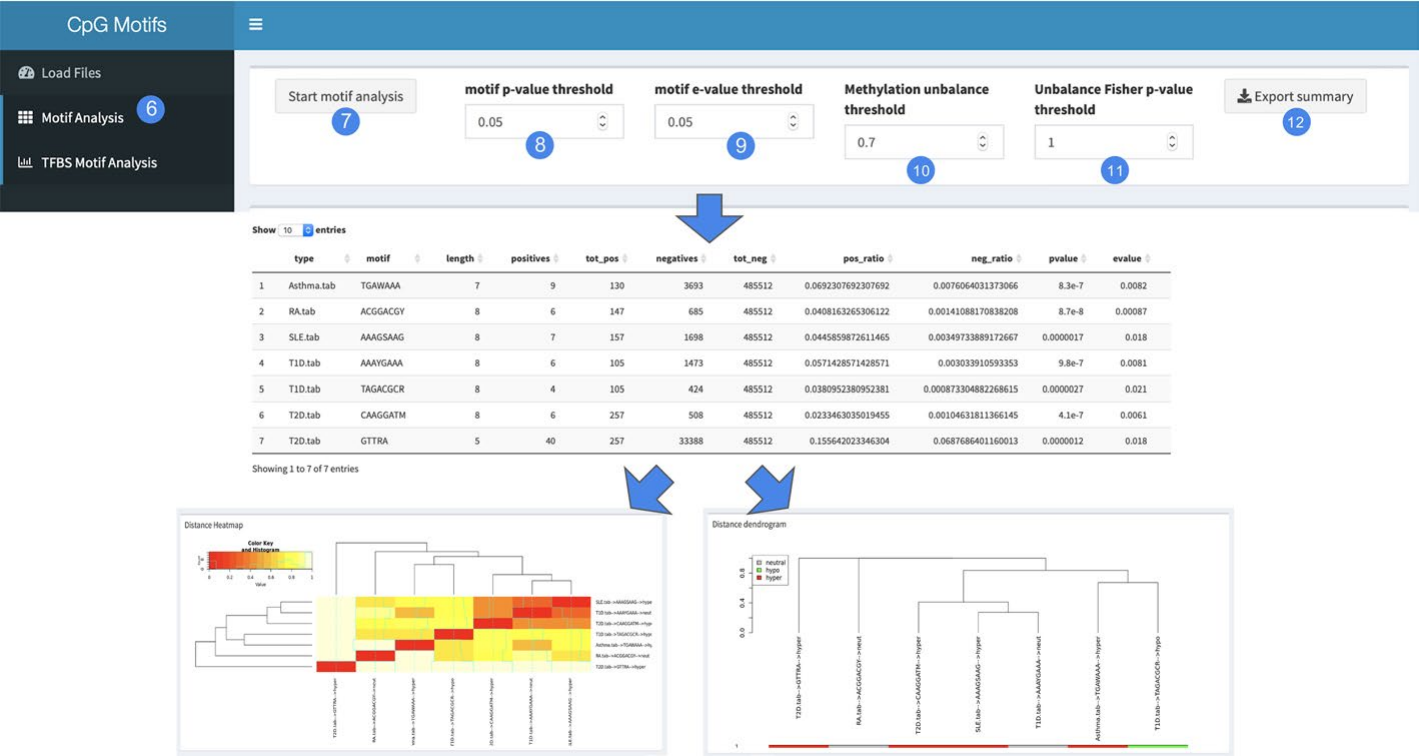
Motif analysis interface of the CpGmotifs tool. Panel A: Parameters setting bar. Panel B: Table reporting the analysis output. Panel C: Heatmap summarizing the analysis results. Panel D: hierarchical clustering of motifs and methylation status annotation bar

The second step of the analysis consists in the detection of enriched CpG motifs, as shown in Fig. 3. The user can perform the analysis by selecting the “Motif analysis” tab (6), where, before starting the motifs search (7), the user has the possibility to set several parameters in order to filter and annotate the results of the motif analysis. In particular, the “motif p-value threshold” field (8) indicates the p-value of the Fisher’s Exact test for the enrichment of the positive motifs over the background sequences. The “motifs e-value threshold” field (9) indicates the motif *p*-value times the number of candidate motifs tested. The “methylation unbalance threshold” field (10) indicates the percentage of methylated or unmethylated CpG in order to consider a certain motif as methylated, unmethylated or neutral. The “Unbalance Fisher *p*-value threshold” refers to the Fisher’s test *p*-value for the enrichment of methylated/unmethylated CpGs in the set of CpG sites carrying the motif. As a result, the tool provides a table reporting all the enriched motifs, including their sequence, length, methylation trend and all the information regarding the statistical tests. By pressing the “Export Summary” button (12) the user can export the results table in .csv format. Along with the table, the tool produces two graphical representations of the results through a heatmap and a clustering dendrogram showing the motifs sequence similarity and their methylation trends. A clustering example is provided in Additional file 1: Figure S1.

The third step of the analysis consists in the identification of putative TFBS overlapping with the significant motifs. Also, in this case the user has the possibility to fine tune the parameters for the statistical analysis and filter the output of the analysis. In detail, the TF *p*-value threshold field (15) indicates the *p*-value threshold of the Tomtom TF enrichment test. The TF e-value threshold (16) indicates the E-value of the Tomtom TF enrichment test. TF q-value threshold (17) indicates the Benjamini & Hochberg corrected *p*-value, while the TF overlap threshold (18) indicates the minimum overlap (in the optimal alignment) of the TFBS with a certain motif to be included in the analysis. The user has the possibility to download the summary table of the obtained results as .csv file (19). Moreover, the user is provided with a heatmap reporting the TFs for which a TFBS has been identified on the columns and the samples on the rows. A transcription factors annotation example is provided in Additional file 1: Figure S2 and Table S2.

**Fig. 4:**
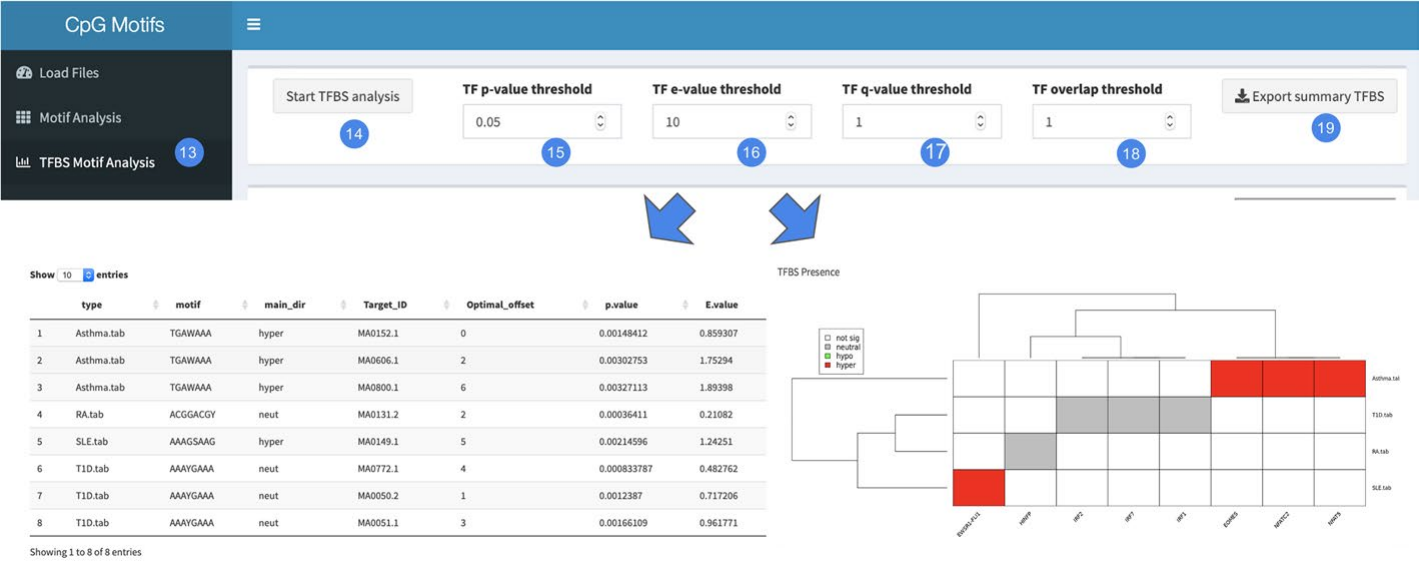
TFBS motif analysis interface of the CpGmotifs tool. Panel A: Parameters setting bar. Panel B: Table reporting the analysis output. Panel C: Heatmap summarizing the analysis results

## Results and discussion

### Case study

We showcase the functionalities of CpGmotifs on five sets of differentially methylated probes in isolated cells from whole blood in selected susceptibility genes for inflammatory diseases, from the catalog of published genome-wide association studies (http://www.genome.gov/gwastudies/) [15], and reanalysed in [16]. The included diseases were asthma, atopic dermatitis, rheumatoid arthritis, systemic lupus erythematosus, type 1 and type 2 diabetes. Through the analysis of these differentially methylated probes we sought to identify whether overrepresented DNA motifs (or motifs families) exist in the context of the deregulated probes. Moreover, we aim at identifying the overall methylation trend of the identified motifs and to infer the regulatory implications of the methylation unbalance through the TFBS prediction.

In order to perform the analysis, the Infinium Human Methylation 450K annotation was chosen. The input to the CpGmotifs tool were 5 target CpG files in tab delimited format (Additional file 1) containing the probes of interest for the five above mentioned inflammatory conditions and the CpG background file containing all the probes reported in the selected annotation. The flanking region size was set to 20. The motif analysis was performed with the following parameters:

- Motif *p*-value threshold: 0.05;
- Motif e-value threshold: 0.05;
- Methylation unbalance threshold: 0.7;
- Unbalance Fisher *p*-value threshold: 1;

We obtained 7 significantly enriched DNA motifs over all the 5 conditions. Of these motifs, 1 was derived from asthma, 1 from rheumatoid arthritis and 1 from systemic lupus erythematosus, while 2 motifs derived from both type 1 and type 2 diabetes. Overall, 4 motifs were characterized as mainly hyper-methylated, 1 motif as hypo-methylated and 2 as neutral. To the best of our knowledge, none of the motifs were previously associated with the relative diseases, meaning that our results could represent an unexplored ground of investigation. The Fig. 3 shows a heatmap based on the sequence similarity of the motifs, while the clustering dendrogram underlines the methylation unbalance trend for each motif.

Subsequently, we the TFBS analysis was performed with the following parameters:

- TF *p*-value threshold: 0.05;
- TF e-value threshold: 10;
- TF q-value threshold: 1;
- TF overlap threshold: 1;

As a result, a total of eight TFBS were found to be significantly associated with the identified DNA motifs (Fig. 4). Of these TFs, three were associated with the motif TGAWAAA deriving from the asthma dataset. Three TFs were associated with the motif AAAYGAAA deriving from type 1 diabetes, while 1 TF is associated with rheumatoid arthritis and 1 from systemic lupus erythematosus. The 3 TFs identified in the asthma dataset are EOMES, NFATC2, and NFAT5 and are known to regulate gene expression during immune responses. In the immune system, Eomes can positively influence the expression of IFN$\gamma$ in CD8+ T-cells [17]. NFAT5 regulates the expression of the TNF$\alpha$, participating in specific aspects of host defense by upregulating genes of the TNF family and other target genes in T cells subjected to osmotic stress [18]. On the other hand, NFATc2 is a key transcription factor to the pathogenesis of allergic responses [19]. It has been demonstrated that its deficiency leads to increased airway hyperresponsiveness (AHR), both after sensitization and in the absence of exogenous allergen challenge. The 3 TFs identified in type 1 diabetes are IRF1, IRF2, IRF7. Aberrations in IRF signaling cascade can lead to increased expression of type I interferon (IFN) genes, IFN-stimulated genes (ISGs), and other pro-inflammatory cytokines/chemokines, leading to the development of numerous diseases, including ones of autoimmune origin, such as type 1 Diabetes. Transcription factor interferon regulatory factor 1 (IRF1), is a downstream target of IFN-gamma/signal transducer and activator of transcription (STAT)-1 and is actively involved in immune-mediated beta cell destruction. It is typically induced by IFN-$\gamma$ via binding of STAT-1 to the IFN-$\gamma$-activation site in the IRF-1 promoter, but other cytokines and hormones can also trigger its expression. IRF-1 is involved in insulin secretion and, especially, in the modulation of chemokine expression by beta cells [20]. IRF2 is a negative regulator of IFN-mediated gene expression. IRF2 suppresses the activity of IRF1 by competing for binding sites. IRF7 has also been implicated in the pathogenesis of type 1 diabetes through the upregulation of inflammatory gene networks [21]. Abnormal expression of Fli-1 is important in the etiology of autoimmune diseases such as systemic lupus erythematosus and systemic sclerosis. Fli-1 is expressed in peripheral blood mononucleated cells and its overexpression correlates with severity of disease in lupus patients [22].

## Conclusions

Here, we presented a novel bioinformatic procedure along with an intuitive graphical interface software to perform the analysis of the sequence context of regions related to DNA methylation events. The comprehension of the relationship linking DNA methylation alterations and proximal DNA sequences is an important step in understanding of the DNA methylation mechanisms and to better identify therapeutic targets in diseases related to methylation aberrations. The technique presented here can be applied to a wide range of public data sets, since it only requires a list of CpGs along with their methylation status. The current implementation supports CpG lists derived from relatively small (hundreds of thousands) set of tested CpGs from the human genome like data derived from the Illumina Infinium 450k or Epic platforms. However, our tool is also able to process CpG lists derived from Whole Genome or Targeted Bisulphite Sequencing. We focused the analysis on CpG methylation events, being the most studied DNA modification, but the analysis can seamlessly be applied to other cytosine modifications in the CHG and CHH contexts.

## Availability and requirements

Project name: CpGmotifs

Project home page: https://github.com/Greco-Lab/CpGmotifs

Operating system(s): Cross Platform

Programming language: R

Other requirements: Docker

License: GPL

Any restrictions to use by non-academics: For commercial use and modifications please contact the corresponding author.

## Supplementary Information

**Additional file 1**. Examples of output results obtained by CpGmotifs analysis.

## Data Availability

All the data used herein are publicly available at http://www.genome.gov/gwastudies.

## Abbreviations

AHR: Airways hyperresponsiveness
DREME: Discriminative regular expression Motif elicitation
GUI: Graphical user interface
IUPAC: International union of pure and applied chemistry
TF: Transcription factor
TFBS: Transcription factor binding site
MEME: Multiple Em for Motif Elicitation
