# Supplementary material for: CpGmotifs: a tool to discover DNA motifs associated to CpG methylation events
Source: BMC Bioinformatics. 2021 May 26;22:278. doi: 10.1186/s12859-021-04191-8 (PMC8157658; doi:10.1186/s12859-021-04191-8)
Supplement: Supplementary file 1 — Additional file 1. Examples of output results obtained by CpGmotifs analysis. [file 12859_2021_4191_MOESM1_ESM.pdf]

Experiment	motif	motif length (bp)	TargetCpGs-seqs with motif	BackCpGs-seqs with motif	TargetCpGs-seqs	BackCpGs-seqs	motif TargetCpGs-seqs ratio	motif BackCpGs-seqs ratio	motif enrich. pvalue	motif enrich. evalue	support set methylation trend
Asthma	TGAWAAA	7	9	130	3693	485512	0.069	0.008	8.3e-07	0.0082	>70% hyper
RA	ACGGACGY	8	6	147	685	485512	0.040	0.001	8.7e-08	0.00087	mixed
SLE	AAAGSAAG	8	7	157	1698	485512	0.045	0.003	1.7e-06	0.018	>70% hyper
T1D	AAAYGAAA	8	6	105	1473	485512	0.057	0.003	9.8e-07	0.0081	mixed
T1D	TAGACGCR	8	4	105	424	485512	0.038	0.001	2.7e-06	0.021	>70% hypo
T2D	CAAGGATM	8	6	257	508	485512	0.023	0.001	4.1e-07	0.0061	>70% hyper
T2D	GTTRA	5	40	257	33388	485512	0.156	0.069	1.2e-06	0.018	>70% hyper

Table S1. Example of motif annotation.

Table showing an example of motifs annotation for motifs derived from 5 groups (Asthma, RA, SLE, T1D, T2D) of differentially methylated CpGs, obtained using Illumina Infinium 450k on whole blood samples (data from https://doi.org/10.1371/journal.pone.0041361 , https://doi.org/10.1371/journal.pone.0041361.s006).
The table reports the experiment name, the enriched motif, the number of bases composing the motif, the number of Target CpGs associated with the motif, the number of CpGs in the background set associated with the motif, the size of the TargetCpG set, the size of the background set, the ratio of CpGs with the motif in the TargetCpG set, the ratio of CpGs with motif in the background set, the motif enrichment p-value as reported by DREME, the motif enrichment e-value as reported by DREME, the trend of hyper- (hypo-) methylation in the Target CpGs associated with the motif.

Table S2. Transcription factors annotation example for motifs reported in Table S1.

Experiment	Motif	p.value	E.value	q.value	Overlap	Orientation	TF
Asthma	TGAWAAA	0,001484	0,859302	1	7	-	NFATC2
Asthma	TGAWAAA	0,003028	1,75294	1	7	-	NFAT5
Asthma	TGAWAAA	0,003271	1,89398	1	7	+	EOMES
Asthma	TGAWAAA	0,008113	4,69726	1	7	-	NFATC3
Asthma	TGAWAAA	0,009673	5,60091	1	7	-	NFATC1
Asthma	TGAWAAA	0,012732	7,37169	1	6	+	ZSCAN4
Asthma	TGAWAAA	0,014175	8,20729	1	7	+	Arid5a
RA	ACGGACGY	0,000364	0,210821	0,421642	8	-	HINFP
RA	ACGGACGY	0,011831	6,8504	1	8	+	ELK1
RA	ACGGACGY	0,011831	6,8504	1	8	+	ELK3
RA	ACGGACGY	0,012166	7,04417	1	8	+	Gabpa
RA	ACGGACGY	0,012166	7,04417	1	8	-	ELK4
RA	ACGGACGY	0,012435	7,1997	1	8	+	ETV1
RA	ACGGACGY	0,013811	7,99636	1	8	+	FEV
RA	ACGGACGY	0,013811	7,99636	1	8	+	ETV4
RA	ACGGACGY	0,013811	7,99636	1	8	+	ETV5
RA	ACGGACGY	0,013888	8,04103	1	8	-	Gmeb1
RA	ACGGACGY	0,014076	8,14986	1	8	+	SPDEF
RA	ACGGACGY	0,01554	8,99742	1	8	+	ELF5
RA	ACGGACGY	0,016354	9,46889	1	8	+	ERG
RA	ACGGACGY	0,016354	9,46889	1	8	+	FLI1
RA	ACGGACGY	0,016354	9,46889	1	8	+	ERF
RA	ACGGACGY	0,017269	9,99882	1	8	+	ETV3
SLE	AAAGSAAG	0,002146	1,24255	1	8	+	EWSR1-FLI1
SLE	AAAGSAAG	0,005404	3,12893	1	7	+	TCF7L2
SLE	AAAGSAAG	0,006399	3,70485	1	8	+	SPIC
SLE	AAAGSAAG	0,007843	4,54116	1	7	+	SPIB
SLE	AAAGSAAG	0,01248	7,22585	1	8	+	IRF3
SLE	AAAGSAAG	0,013024	7,54079	1	8	+	ELK3
SLE	AAAGSAAG	0,013807	7,99438	1	8	+	ELK1
SLE	AAAGSAAG	0,013807	7,99438	1	8	+	FEV
SLE	AAAGSAAG	0,013807	7,99438	1	8	+	ERF
SLE	AAAGSAAG	0,014788	8,56221	1	8	+	SPI1
SLE	AAAGSAAG	0,015589	9,02587	1	8	+	ETV4
T1D	AAAYGAAA	8,44E-06	0,004888	0,009777	8	-	STAT1::STAT2
T1D	AAAYGAAA	0,000834	0,482756	0,47813	8	+	IRF7
T1D	AAAYGAAA	0,001239	0,717196	0,47813	8	-	IRF1
T1D	AAAYGAAA	0,001661	0,96176	0,48088	8	+	IRF2
T1D	AAAYGAAA	0,005561	3,2196	1	8	+	SIX2
T1D	AAAYGAAA	0,005909	3,42114	1	7	-	POU2F2
T1D	AAAYGAAA	0,006676	3,86568	1	8	+	Pou2f3
T1D	AAAYGAAA	0,008578	4,96655	1	8	+	IRF9
T1D	AAAYGAAA	0,008671	5,02066	1	8	+	IRF8
T1D	AAAYGAAA	0,009472	5,48445	1	8	+	IRF5
T1D	AAAYGAAA	0,011728	6,79027	1	8	+	IRF4
T1D	AAAYGAAA	0,013368	7,74008	1	8	+	IRF3
T1D	AAAYGAAA	0,014573	8,43746	1	7	-	NFATC3
T1D	TAGACGCR	0,008271	4,78864	1	6	-	SMAD3
T1D	TAGACGCR	0,01262	7,30672	1	6	-	Ahr::Arnt
T2D	CAAGGATM	0,013738	7,95448	1	8	+	NR2F1
T2D	CAAGGATM	0,014649	8,48188	1	8	+	NR1H4
T2D	GTTRA	0,009903	5,73385	1	5	+	MYBL1
T2D	GTTRA	0,011134	6,44658	1	5	+	HNF1B
T2D	GTTRA	0,013008	7,53132	1	5	-	MEOX2
T2D	GTTRA	0,013591	7,86938	1	5	-	HNF1A
T2D	GTTRA	0,01375	7,96107	1	5	-	NR1H2::RXRA

T2D	GTTRA	0,015794	9,14459	1	5	+	HMBOX1
T2D	GTTRA	0,015794	9,14459	1	5	-	ESX1

The table shows JASPAR TFs reported by the TOMTOM tool using the motifs in Table S1.
For each pair of motif-TF, the following information is reported: the experiment name, the motif's sequence, the match p-value, e-value and q-value, the length of the detected overlap between the motif and the TF binding sequence, the DNA orientation of the overlap, the TF id.

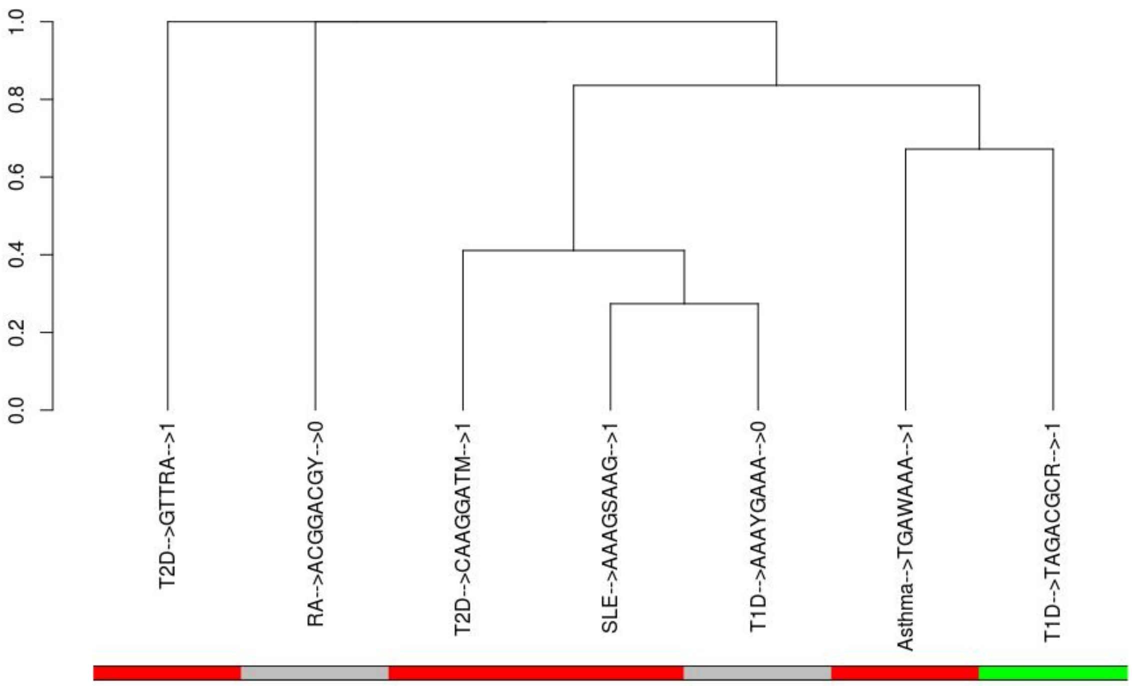

Figure S1. A motif clustering example using data from Table S1. Hierarchical clustering has been obtained applying Ward method. DNA pairwise similarity between motifs, normalizing it in the range [0 ,1] using the min-max normalization and complementing it to 1 to get a distance measure. Dendrogram labels report the following information separated by the symbol "-->" : the experiment name, the motif sequence and the methylation trend (coded as 1 if more than 70% of CpGs in the support set were hyper-methylated, -1 if more than 70% of CpGs in the support set were hypo-methylated, 0 otherwise). Lower bar indicates the methylation trend in the motif support set.

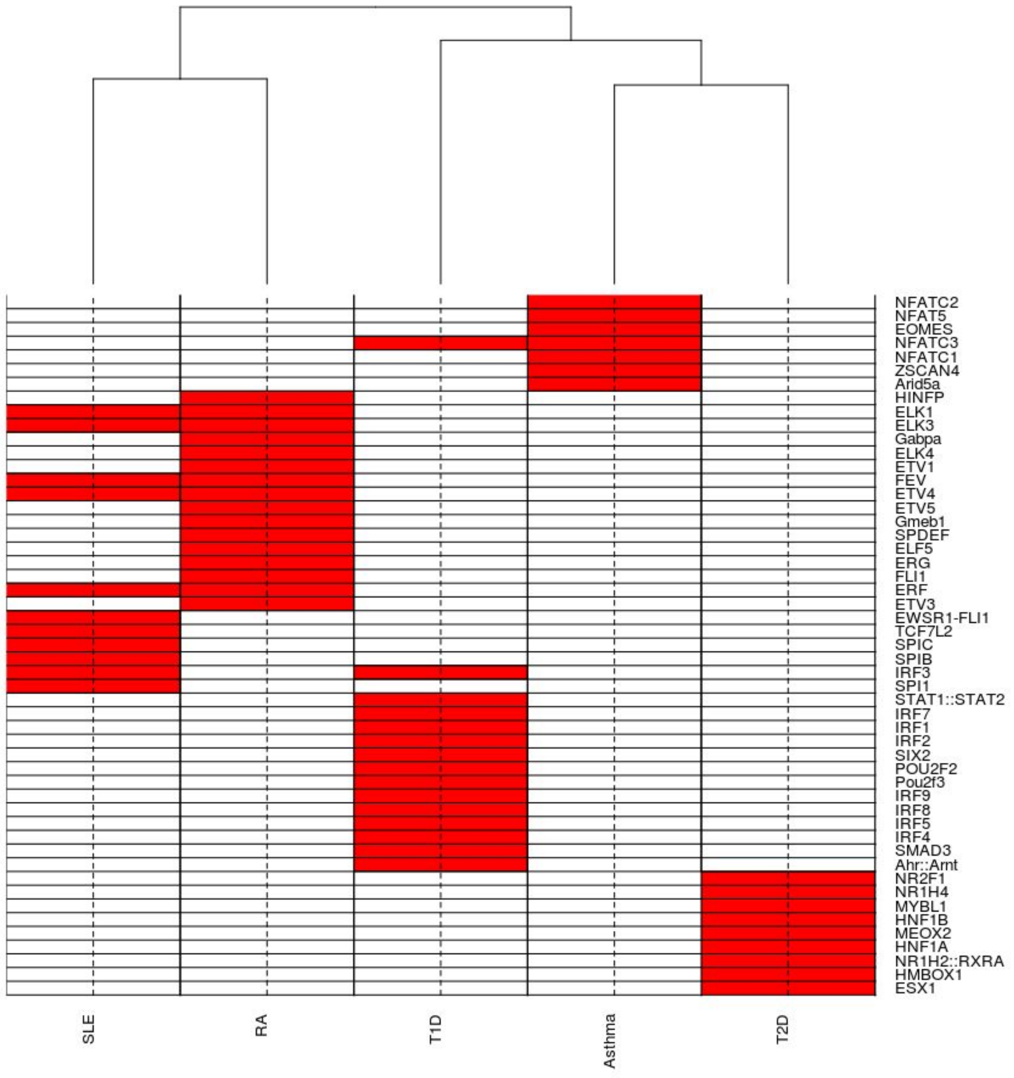
NFATC2
NFAT5
EOMES
NFATC3
NFATC1
ZSCAN4
Arid5a
HINFP
ELK1
ELK3
Gabpa
ELK4
ETV1
FEV
ETV4
ETV5
Gmeb1
SPDEF
ELF5
ERG
FLI1
ERF
ETV3
EWSR1-FLI1
TCF7L2
SPIC
SPIB
IRF3
SPI1
STAT1::STAT2
IRF7
IRF1
IRF2
SIX2
POU2F2
Pou2f3
IRF9
IRF8
IRF5
IRF4
SMAD3
Ahr::Arnt
NR2F1
NR1H4
MYBL1
HNF1B
MEOX2
HNF1A
NR1H2::RXRA
HMBOX1
ESX1
SLE
RA
T1D
Asthma
T2D

Figure S2. Transcription factors annotation example for motifs presented in Table S1.
Enriched TFs are reported in rows, experiments are reported in columns.
Red cells indicate that the corresponding TF shows affinity for one or more enriched motifs in the corresponding experiment.
